# Effects of 12-Year Nitrogen Addition and Mowing on Plant-Soil Micronutrients in a Typical Steppe

**DOI:** 10.3390/plants11223042

**Published:** 2022-11-10

**Authors:** Guoxiang Niu, Yinliu Wang, Guangyi Dai, Siwei Xie, Yiqian Jin, Junjie Yang, Jianhui Huang

**Affiliations:** 1 Key Laboratory of Vegetation Restoration and Management of Degraded Ecosystems, South China Botanical Garden, Chinese Academy of Sciences, Guangzhou 510650, China; 2 South China National Botanical Garden, Guangzhou 510650, China; 3 State Key Laboratory of Vegetation and Environmental Change, Institute of Botany, Chinese Academy of Sciences, Xiangshan, Beijing 100093, China; 4 Opening public laboratory, South China Botanical Garden, Chinese Academy of Sciences, Guangzhou 510650, China; 5 College of Sciences, University of Strathclyde, Glasgow G4 0LZ, UK; 6 International department, High School Affiliated to South China Normal University, Guangzhou 510650, China

**Keywords:** nitrogen deposition, mowing, micronutrient cycling, plant nutrition, Mongolian Plateau

## Abstract

Changes in soil micronutrient availability may have adverse consequences on grassland productivity, yet it’s still largely unclear how concurrent human practices, such as fertilization and mowing, affect micronutrient cycling in the plant-soil systems. Here, we measured six essential micronutrient (Fe, Mn, Cu, Zn, Co and Mo) contents in both plant pool (separated as aboveground plant parts, litter, and belowground roots) at the community level and soil pool (0–10 cm depth) after 12-year consecutive nitrogen (N) addition (0, 2, 10, and 50 g N m^−2^ year^−1^) and mowing in a typical steppe of the Mongolian Plateau. The results show that (i) medium-N (10 g m^−2^ year^−1^) and high-N (50 g m^−2^ year^−1^) addition rates significantly increased contents of soil-available Fe (+310.0%, averaging across the two N addition rates), Mn (+149.2%), Co (+123.6%) and Mo (+73.9%) irrespective of mowing treatment, whereas these addition treatments usually decreased contents of soil total Fe (−8.9%), Mn (−21.6%), Cu (−15.9%), Zn (−19.5%), Co (−16.4%) and Mo (−34.7%). (ii) Contents of Fe in aboveground plant parts, litter, and roots significantly decreased, whereas plant Mn increased with N addition. Contents of above ground plant Cu, Zn, Co, and Mo significantly decreased at high-N addition rate, whereas contents of micronutrients in roots and litters, except for Fe, generally increased with N addition. Moreover, the total amount of micronutrients in the plant pool (contents × biomass) significantly increased at the medium-N addition rate but decreased at the high-N addition rate. All N addition rates significantly enlarged the pool of litter micronutrients, and roots could hold more micronutrients under N addition, especially combined with mowing treatment. Importantly, although mowing could regulate the effects of N addition on variables (i) and (ii), the effects were weaker overall than those of N addition. (iii) Changes in root micronutrients, except for Mn, could explain corresponding changes in plant micronutrients (R^2^: 0.19–0.56, all *p* < 0.01), and significant linear correlations were also observed between soil-available Fe and Fe in plant and roots. Aboveground plant Mn was significantly correlated with soil-available Mn, while Co and Mo in roots were also significantly correlated with soil-available Co and Mo. These results indicate that soil micronutrient supply capacity may decrease due to a decrease in total micronutrient contents after long-term N addition and mowing. They also suggest that different magnitude responses of soil micronutrients in plants (i.e., litters, roots) and soil should be considered when comprehensively examining nutrient cycling in grassland ecosystems.

## 1. Introduction

Biogeochemical cycling of micronutrients in pasture systems has received much attention in recent years [1,2,3]. Inadequate micronutrients may affect plant growth and limit community productivity [4,5], whereas excessive soil micronutrients can be toxic to plants and threaten ruminant health [6]. For instance, most metal micronutrients, such as iron (Fe), manganese (Mn), Copper (Cu), zinc (Zn) and molybdenum (Mo), are critical enzyme cofactors and catalyzing diverse reactions in plant processes [7,8], whereas Mn plant toxicity frequently occurs in low pH soil [9]. Although Cobalt (Co) is not considered a nutrient necessary for plant growth, it can help plants assimilate more nitrogen (N) in N-limited systems [10]. Importantly, micronutrients influence plant capacity against biotic and abiotic constraints in grassland ecosystems [3,11]. Nevertheless, compared with other mineral nutrients, comprehensively examining the role of micronutrients in grassland productivity has been challenging because of the small number of relevant studies.

Atmospheric N deposition has increased at an incredible rate because of the overconsumption of fossil fuels and fertilizers, which can significantly impact biogeochemical cycling of micronutrients in grassland ecosystems [3,12]. On the one hand, excessive N inputs usually increase the primary productivity in most grassland ecosystems because of their alleviation of N limitations [13]. In this respect, the plant community assimilates more of the other nutrients, including micronutrients, to maintain a high plant biomass. To ensure an adequate supply of micronutrients and avoid toxicity, plants optimize the required uptake of different micronutrients from soil. They also optimize micronutrient distribution throughout the plant, allocate them to specific metalloproteins, and deliver them to sink organs [1,14]. On the other hand, N addition may also alter soil physicochemical properties (e.g., a decrease in soil pH), and further affect the hydrolysis of micronutrients [15]. Moreover, N addition may impact the return of micronutrients from litters to soil due to the restrained decomposition of litter [16,17]. In other words, the plant pools (i.e., aboveground plant parts, litter, and belowground roots) of micronutrients with N addition might have increased, but this point still requires verification. Nevertheless, research on elemental micronutrient supply in pasture systems has focused less on changes in the leaves, litter, roots and soil with N addition. Less is therefore known about changes in some important micronutrients, such as Mo and Co in grassland ecosystems, because most studies did not detect their changes.

Mowing, a common grassland management practice, has become more important because it not only provides hay and grassland-related products to humans, but also prevents grassland degradation caused by heavy grazing [18,19]. However, mowing could also affect the availability of soil micronutrients in different ways. For instance, mowing can remove a certain amount of micronutrient from the ecosystem by directly reducing the amounts of standing materials on the ground, but these lost nutrients cannot be replenished in a timely manner. It can also directly influence the microclimate, such as reducing soil moisture and increasing soil surface temperature, thus subsequently affecting plant growth and microbial activities [20,21]. Importantly, the combined effects of N addition with mowing on ecosystem structure and function are inconsistent with their individual effects [22]. In grassland ecosystems, mowing usually enhances the positive effects of N addition on water use efficiency and ecosystem carbon fluxes [20], but mitigates the negative effects on plant species diversity [22]. A recent study in meadow steppe ecosystem also found, in a three-year field experiment, that the effects of N addition effects on Fe, Mn, Cu and Zn in plants and soil depend on the N form and mowing management [23]. Nevertheless, most previous studies have usually considered a short treatment of N addition and mowing management (≤6 years), and few of these focused on changes in micronutrients in both plants and soil.

This study investigates the responses of micronutrients in plants versus soil to N addition and mowing in the steppe grassland in northern China. As an essential part of the Eurasian steppe, this grassland has been praised as the “Paradise Grassland”, with important economic value and ecological significance [24]. However, they confront some extrinsic factors, such as increasing N deposition and mowing. The ways that these factors impact ecosystem micronutrient cycling are uncertain. A 12-year (2008–2020) field experiment was performed to test three hypotheses as follows: (1) N addition would increase the availability of soil micronutrients with increasing plant uptake; (2) such effects were also regulated by mowing management; and (3) N addition does not affect the micronutrient pool size (content × dry biomass) in aboveground plant parts, litter, and belowground roots at community level and soil, but mowing decreases their micronutrient pool size because of the clipping of the aboveground plant biomass.

## 2. Results

### 2.1. Soil-available and Total Micronutrient Content

**Figure 1:**
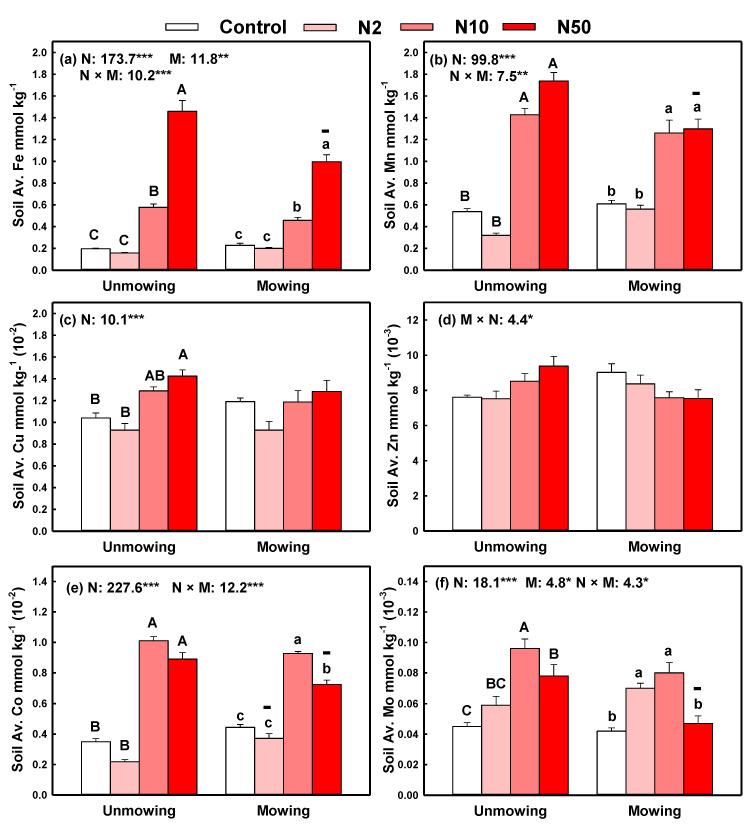
Effects of N addition on the contents of soil-available Fe, Av. Fe; available Mn, Av. Mn; available Cu, Av. Cu; available Zn, Av. Zn; available Co, Av. Co and available Mo, Av. Mo with unmowing and mowing treatments. Note: Data are presented as means ± standard error. The results of two-way ANOVA (F values) are shown at the top of each figure. *, **, *** indicates significant differences at *p* < 0.05, 0.01, 0.001, respectively. Different uppercase or lowercase letters denote significant differences (*p* < 0.05) between control and N addition plots with unmowing and mowing treatments. Symbols +, − indicate significant positive or negative effects of mowing compared with their counterparts with the same N addition rate at *p* < 0.05. Only items with significant cases were shown.

The contents of soil-available Fe and Mo were significantly affected by N addition, mowing, and their interaction. Soil-available Fe significantly increased under N10 and N50 treatments regardless of mowing treatment (Figure 1a,f). Soil-available Mo also significantly increased under N10 and N50 treatments in unmown plots and under N2 and N10 treatments in mown plots (Figure 1f). Contents of soil-available Mn and Co were affected by N addition and its interaction with mowing (*p* < 0.05), while their contents significantly increased under high N inputs (N10 and N50) in both mown and unmown plots (Figure 1b,e). High N addition (N50) also increased the soil-available Cu content in unmown plots (Figure 1c), while N addition had no significant effects on soil-available Zn content in both mown and unmown plots (Figure 1d). However, for the total micronutrient content, N addition significantly decreased the contents of soil total Fe by 5.7% (averaging across the three N addition rates in both mown and unmown plots), Mn by 15.9%, Cu by 12.1%, Zn by 14.2%, Co by 11.9%, and Mo by 23.9 (Figure S1). These decreasing effects were generally significant under N10 and N50 treatments (Figure S1). In addition, mowing and N10 treatment (i.e., N10+Mowing) significantly decreased the contents of soil-available Fe, Mn, Co and Mo as compared with those of the same N addition rates (i.e., N10), and low-N addition also decreased soil-available Co content (Figure 1).

### 2.2. Micronutrients in Aboveground Plant Parts, Litter, and Belowground Roots

**Figure 2:**
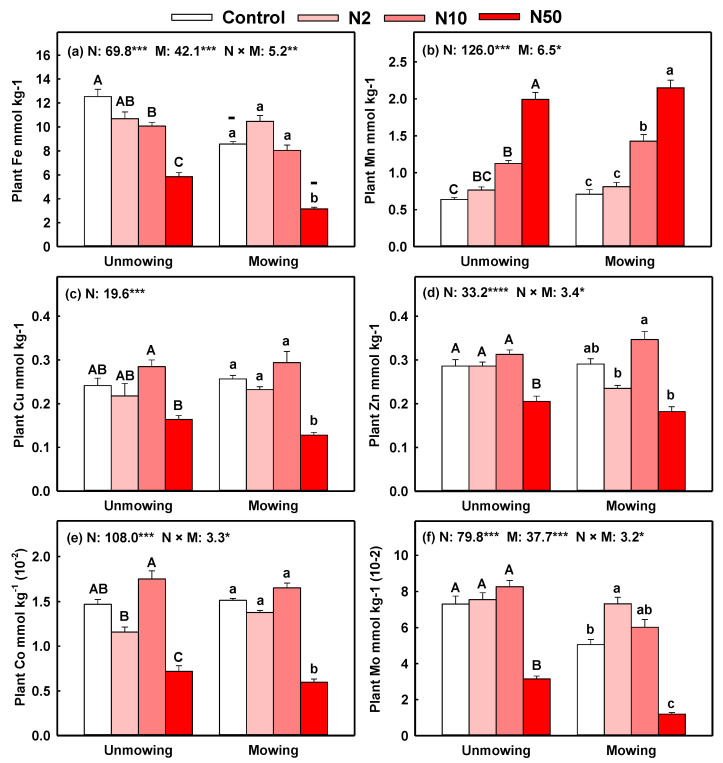
Effects of N addition on the contents of six micronutrients (Fe, Mn, Cu, Zn, Co, and Mo) with unmowing and mowing treatments in aboveground plant parts. Note: Data are presented as means ± standard error. The results of two-way ANOVA (F values) are shown at the top of each figure. *, **, *** indicates significant differences at *p* < 0.05, 0.01, 0.001, respectively. Different uppercase or lowercase letters denote significant differences (*p* < 0.05) between control and N addition plots with unmowing and mowing treatments. Symbols +, − indicate significant positive or negative effects of mowing compared with their counterparts with the same N addition rate at *p* < 0.05. Only items with significant cases were shown.

The contents of Fe and Mo in aboveground plant parts were affected by N addition, mowing, and their interaction, while contents of plant Mn, Zn, and Co were affected by N addition and its interaction with mowing (*p* < 0.05, Figure 2). Specifically, plant Fe significantly decreased with increasing N addition rates regardless of mowing, whereas plant Mn significantly increased with N addition rates (Figure 2a,b). High N addition (N50) significantly decreased plant Co and Mo in both mown and unmown plots, but low N addition (N2) significantly increased plant Mo content in mown plots (Figure 2e,f). High N addition also significantly decreased plant Zn in unmown plots, and plant Cu in mown plots (Figure 2a,d). However, for plant pools (content × dry biomass) of these six micronutrients, N addition significantly increased the pools of plant Mn regardless of mowing (Figure S2b). The plant pools of Fe, Cu, Zn, Co and Mo were usually increased with N2 and N10 treatments but decreased with N50 treatment (Figure S2).

**Figure 3:**
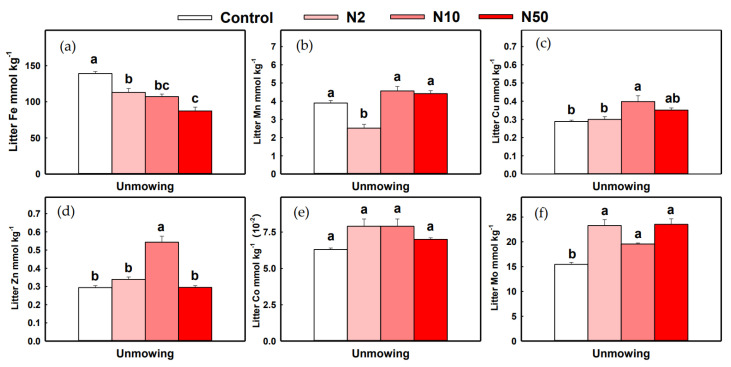
Effects of N addition on the contents of six micronutrients (Fe, Mn, Cu, Zn, Co, and Mo) with unmowing treatments in litter. Notes: Different capital letters denote significant differences (*p* < 0.05) between control and N addition plots. (Fe, Mn, Cu, Zn, Co, and Mo (**a**–**f**)).

In unmown plots, litter Fe content significantly decreased with increased N addition rates, whereas litter Zn content significantly increased with N addition (Figure 3a,f). Litter Mn content decreased with N2 treatment, and litter Cu content increased with N10 treatment (Figure 3b,c). However, no consistent pattern of litter pools of these micronutrients was observed. The litter pools of all micronutrients were significantly increased with all N addition rates, except for litter Mn under N2 treatment (Figure S3).

**Figure 4:**
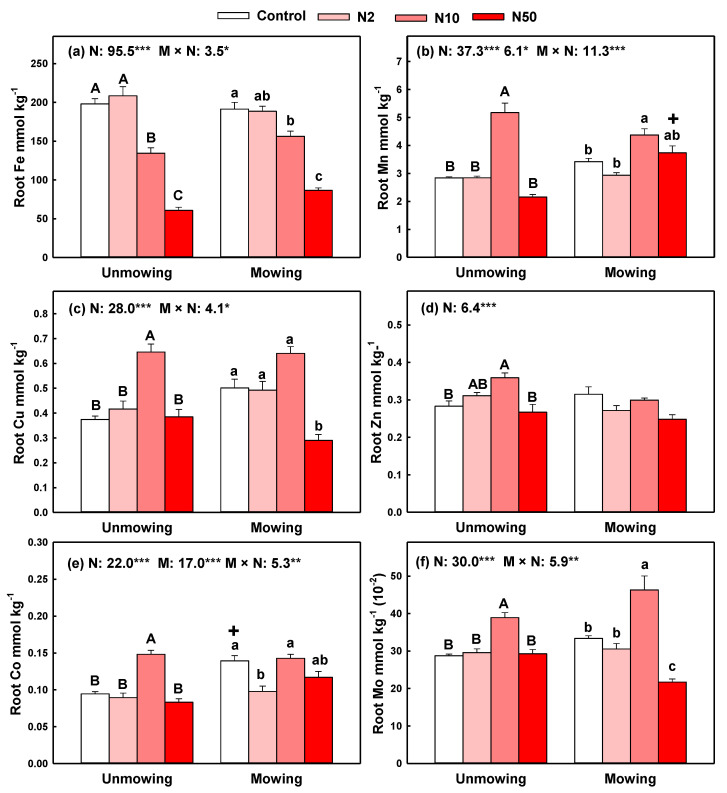
Effects of N addition on the contents of six micronutrients (Fe, Mn, Cu, Zn, Co, and Mo) with unmowing and mowing treatments in belowground roots. Note: Data are presented as means ± standard error. The results of two-way ANOVA (F values) are shown at the top of each figure. *, **, *** indicates significant differences at *p* < 0.05, 0.01, 0.001, respectively. Different uppercase or lowercase letters denote significant differences (*p* < 0.05) between control and N addition plots with unmowing and mowing treatments. Symbols +, − indicate significant positive or negative effects of mowing compared with their counterparts with the same N addition rate at *p* < 0.05. Only items with significant cases were shown.

The content of Co in belowground roots was also affected by N addition, mowing and their interaction, while contents of litter Fe, Mn, Cu and Mo were affected by N addition and its interaction with mowing (Figure 4). Medium and High N addition significantly decreased root Fe content, whereas medium N addition significantly increased root Mn, Cu and Co contents in both mown and unmown plots (Figure 4). Medium N addition also significantly increased root Zn content in unmown plots (Figure 4d). At the same N addition levels, mowing significantly decreased root Mn content as compared with that in unmown plots, but increased root Co content (Figure 4b,e). Additionally, N addition significantly decreased root Fe pools in unmown plots, whereas N addition, especially under N10 and N50 treatments in both mown and unmown plots, generally increased the pools of root Mn, Cu, Zn, Co and Mo (Figure S4).

### 2.3. Correlations between Soil and Plant Micronutrients

**Figure 5:**
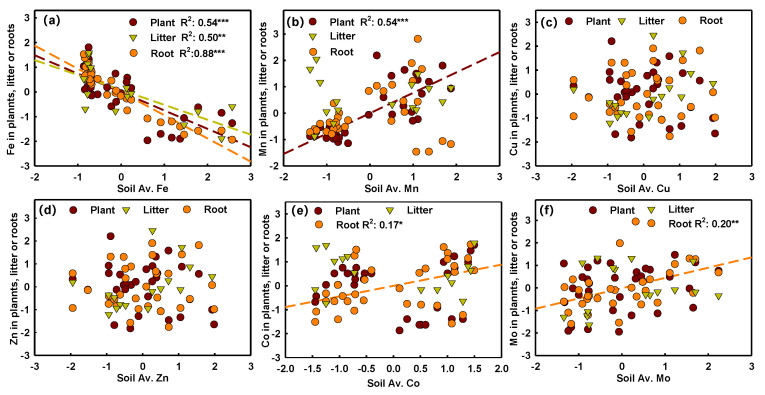
Relationships of soil-available Fe, Mn, Cu, Zn, Co, and Mo with their corresponding contents in aboveground plants (purple points), litter (green triangles) and roots (orange points). Note: The results (R^2^ and *p* value) of linear regression are shown at the top of each figure using the corresponding color and symbols. *, **, *** indicates significant linear relationship at *p* < 0.05, 0.01, 0.001, respectively. Only significant items are shown.

**Figure 6:**
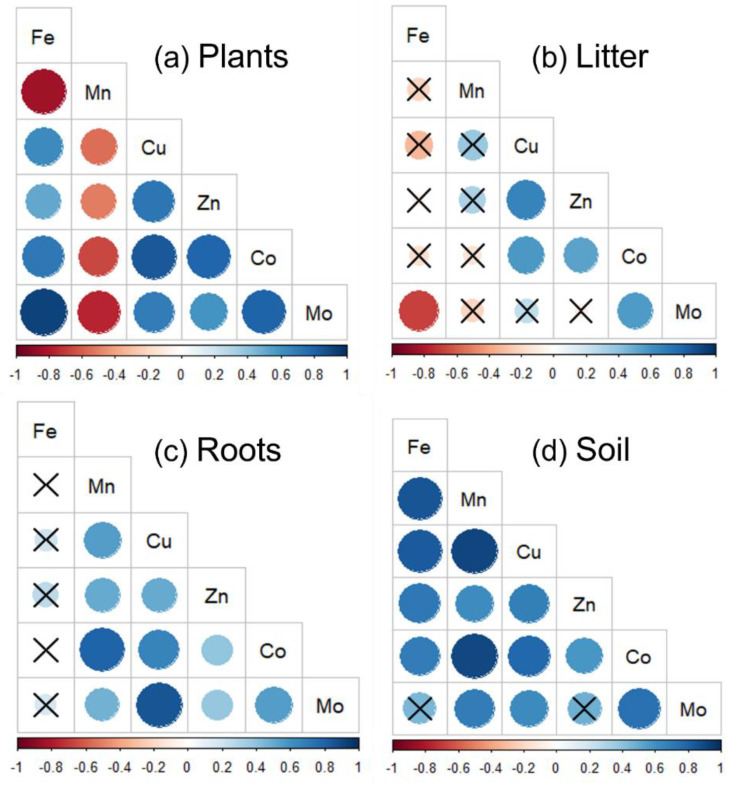
Pearson correlations among six micronutrients (Fe, Mn, Cu, Zn, Co, and Mo) in aboveground plants (**a**), litter (**b**), roots (**c**) and soil (**d**). Red circles indicate positive correlations, whereas bule circles indicate negative correlations, and the symbol × indicates insignificant correlations.

Soil-available Fe was significantly negatively correlated with Fe in plants, litter, and roots, and it explained 54%, 50%, and 88% of total variations, respectively (Figure 5a). Soil-available Mn was significantly positively correlated with plant Mn (R^2^: 0.54), while soil-available Co and Mo were significantly positively correlated with root Co (R^2^: 0.17) and Mo (R^2^: 0.20), respectively (Figure 5b,e,f). However, the relationships of soil total Fe and Mn with each plant part were opposite to those of soil-available Fe and Mn with each plant part (Figure S5a,b). Moreover, soil total Cu was negatively correlated with litter Cu (R^2^: 0.30), whereas soil total Mo was positively correlated with Mo in plants (R^2^: 0.22) and roots (R^2^: 0.08) (Figure S5c,f). Additionally, significant negative correlations of soil-available Mn with the other available five micronutrients, and soil-available Fe with available Mo, were only observed in aboveground plant and litter, respectively (Figure 6).

## 3. Materials and Methods

### 3.1. Study Site

This study was conducted in a steppe grassland site at the Inner Mongolia Grassland Ecosystem Research Station (IMGERS) in northern China (43°32′45″ N, 116°40′30″ E). Based on meteorological records from IMGERS (1985–2019), the study area has a semiarid continental climate, with a mean annual temperature of about 1.0 °C, and mean annual precipitation of about 321.9 mm. More than 70% of precipitation occurs during the growing season from June to August. The soil is a Haplic Calcisol soil (FAO soil classification system), comprising soil silt and clay at 44.2% and 5.2% in 0–10 cm layer, respectively [25]. The background annual N deposition rate was less than 2 g m^−2^ year^−1^, and no fertilizers were applied to the study site until the start of this experiment [26].

### 3.2. Experimental Design and Sampling

**Figure 7:**
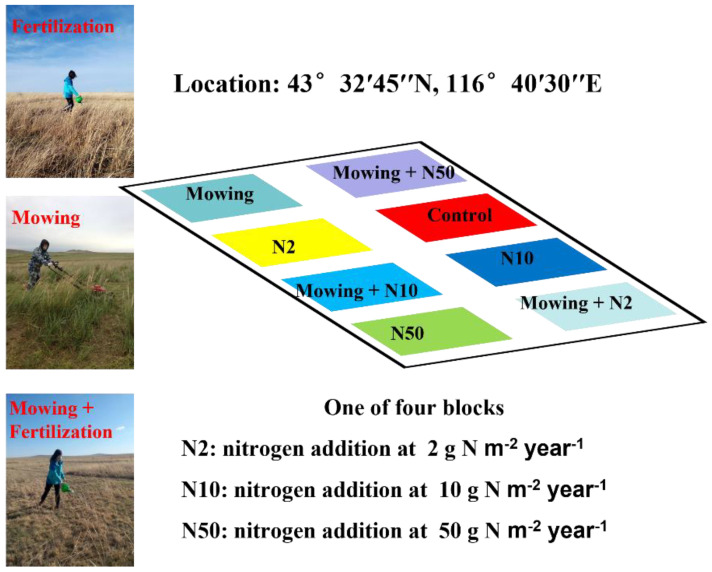
Map of the nitrogen addition and mowing treatment plots.

To exclude large animal grazing and other human interference, the study site was fenced in 1999 and field treatments began in 2008. The experiment was arranged in a completely randomized block design with ten blocks, and each block contained 38 plots (8 m × 8 m) with a 40-cm buffer around each. Detailed experimental information can be found in our previous studies [25,27]. Briefly, four N treatments (0, 2, 10 and 50 g N m^−2^ year^−1^, representing control, low N, medium N, and high N addition, respectively) with or without mowing management were selected in this study (Figure 7). We also had a total of eight treatment combinations, including Control, N2, N10, N50, Control+Mowing, N2+Mowing, N10+Mowing, N50+Mowing. Each treatment combination had four replicates resulting in 32 plots.

Ammonium nitrate (NH_4_NO_3_) was annually added in equal amounts on the first day of June and November. Mowing treatment was conducted by clipping the aboveground vegetation at a 10-cm height above soil surface once a year in late August and the harvested materials were removed immediately.

In August 2020 (i.e., after 12 years of N addition treatments), we collected samples of both plant (above-ground plants, litter and roots) and soil in each plot before mowing treatment. Above-ground plant parts were clipped from three random quadrats (0.25 m × 0.25 m) in each plot from the four blocks, and litter on the surface soil was also collected by hand. No litter was collected in mowed plots because most biomass was clipped. After removing the litter on the surface soil, root and soil samples were collected from each quadrat using a 6.8 cm diameter corer and were sieved to 2-mm. The part that passed through (<2 mm) was collected as the soil sample, while the part remaining on the sieve was used as root sample. All samples from three quadrats were mixed into one sample. All plant materials were washed under tap to remove mixed soil and stones. Then, these samples were dried to constant weight in a 60 °C oven and further weighted. Soil samples were air-dried, ground and further analyzed for micronutrient concentration.

### 3.3. Soil and Plant Measurements

The content of soil-available Fe, Mn, Cu, Zn, and Co was measured using diethylenetriaminepentadcetic (DTPA) method [27,28]. Briefly, 15 g air-dried soil (<2 mm) was extracted with a 45 mL mixture solution of 0.1 mol L^−1^ triethanolamine, 0.01 mol L^−1^ CaCl_2_ and 0.005 mol L^−1^ DTPA acid at pH 7.3. The mixture was shaken at 200 rpm for 2 h and filtered through a filter paper, and the content of these five available micronutrients was determined using inductively coupled plasma optical emission spectrometer (ICP-OES, Thermo Electron Corporation, Waltham, Massachusetts, USA). Soil-available Mo was extracted with a mixture solution of 0.15 mol L^−1^ oxalic acid and 0.20 mol L^−1^ ammonium oxalate at pH 3.3 [29,30]. The mixture was also shaken, filtered and measured by ICP-OES. Soil total Fe, Mn, Cu, Zn, Co, and Mo content were digested using a strong acid mixture solution (including 10 mL HNO_3_ + 2 mL HF + 2 mL HClO). Approximately 500 mg of ground soil was weighted and digested with a heating tube. After digestion, the solution was filled to a volume of 15 mL and was measured by ICP-OES.

To determine the total contents of Fe, Mn, Cu, Zn, Co, and Mo in plants, litter and roots, a strong acid mixture solution (including 8 mL HNO_3_ + 2 mL HF) was used to digest plant parts [31,32]. After weighing, biomass samples of plants, litter and roots in each plot were cut into small pieces and mixed uniformly. All litter and root materials were ground in a ball mill, whereas only a 50 g aboveground plant was ground because of its high biomass. The digested solution was also measured by ICP-OES. The units of all micronutrients in both plant parts and soil were converted to mmol kg^−1^. Moreover, the pool size of the three plant parts was calculated using the micronutrient content multiplied by sample biomass (content × dry biomass), and the units were converted to mmol m^−2^.

### 3.4. Statistical Analyses

All statistical analyses were performed in R v4.1.3 with significance set to *p* < 0.05. Two-way ANOVA was used to evaluate the individual and interactive effects of N addition and mowing treatments on six micronutrients (Fe, Mn, Cu, Zn, Co, and Mo) in aboveground plant parts, litter, below ground roots and soil. One-way ANOVA with Tukey’s honestly significant difference test was employed to further assess the effects of different N addition rates on these six micronutrients in mowed or unmown plots. Linear regressions were executed to identify the relationships of micronutrient in three plant parts with the soil-available and total micronutrient contents. Pearson correlation analyses were also performed to explore the potential correlations of micronutrients in both plant parts and soil.

## 4. Discussion

### 4.1. Effects of N Addition on Micronutrients in the Plant-Soil System

Inconsistent with the first and third hypotheses, the significant increase in soil-available micronutrients could not always promote the micronutrient uptake in all plant parts, and N addition affected the micronutrient pool size in aboveground plant parts, litter, and belowground roots and soil. For instance, although soil-available micronutrients (except for Zn) significantly increased with N addition, significant increase in Mn was only observed in plants and roots, and the contents of most micronutrients increased with N addition only in either one of the plants, litter, or roots. Moreover, the significant N addition effects of micronutrients varied with plants, litter, and roots, and these effects depended on N addition rates. Meanwhile, we found that soil total micronutrient pool decreased, and more micronutrients could be stored in plants, litter, or roots.

The significant increase in soil-available micronutrients might be correlated with the decrease in soil pH, because low soil pH under N addition can accelerate hydrolysis process (e.g., the dissolution of Mn oxides and Fe hydroxides), and most micronutrients can be released from rocks [33,34]. Correspondingly, we found that soil pH significantly decreased at N10 and N50 treatments in this study, while soil organic carbon content generally increased with N addition (Table S1). By contrast with soil-available micronutrients, soil total micronutrients (except for total Fe) significantly decreased with N addition, and two reasons may help us understand this phenomenon. First, N addition significantly increased plant community biomass in this N-limiting ecosystem and enhanced plant total micronutrient uptake [35,36]. In other words, plant parts store more micronutrients under N addition saturation because these micronutrients in plants were not inputted into the soil in a timely manner by microbial decomposition processes. This point is supported by the high amounts of accumulated micronutrients in litters (Figure S3). Previously, we have also reported that only long-term (10-year) N addition increased micronutrients (Fe, Mn, Cu, Zn) by an average of 20.4% in topsoil (0–20 cm), with no pronounced effects of short-term (2-year) N addition being observed [27]. Second, more soil micronutrients might be lost via leaching processes, especially under high N addition rates, because of high contents of soluble N in N addition plots being observed [25,37]. These observations may suggest that long-term N addition can deplete soil micronutrient pools especially in the topsoil, and that more studies should focus on whether high contents of micronutrients in plants impacts forage quality.

In addition, the response of Fe in the three plant parts to N addition was inconsistent with the responses of the other five micronutrients. The results suggest that the amount of absorption of a certain element by plants was affected not only by the concentration of this element in soil, but also by the concentration of the other elements [1,36]. For instance, some studies have indicated that the uptake of Mn in plants could be inhibited if plants absorb excessive Mn [9,36,38]. This point was further evidenced by the significant negative correlations of Fe with the other five micronutrients in plants and litter (Figure 6). Additionally, we found that N addition rate was an important moderator because the responses of micronutrients in both plant parts and soil varied with N addition levels. Low N addition rates (≤ 10 g m^−2^ yr^−1^) positively affected plant community products or microbial activities via alleviation of their N limitations, whereas high N addition rates caused adverse effects due to soil acidification [25]. Inconsistent with the third hypotheses, we further found that the distribution of micronutrients in different plant parts was altered after long-term N addition, and that roots and litter could store more micronutrients than aboveground plant parts. These findings suggest that plants reallocate micronutrients throughout the body and deliver them to sink parts (i.e., roots and litter) to ensure a sufficient nutrient supply or avoid toxicity under N addition because of aboveground live plant parts (i.e., leaves and stems) being more vulnerable to stresses [8,39,40,41].

### 4.2. Effects of Mowing and N Addition on Micronutrients in the Plant-Soil System

Consistent with the second hypotheses, mowing could regulate the N addition effects on micronutrients in the plant–soil system. However, such regulating effects were weak, and N addition played a leading part in all cases because of the lower *F* values and higher *p* values of mowing effects than those of N addition effects (Figure 1, Figure 2 and Figure 3). In addition, mowing did not decrease the micronutrient pool size in three plant parts but decreased soil micronutrient pools. Compared with the control plots, mowing alone had no significant negative effects on micronutrients in both plants and soil in the present study. This lack of negative effect might be because long-term mowing stimulated plant growth and because roots absorb more micronutrients from deep soil [18,22,42]. 

Some previous studies have demonstrated that mowing can strongly affect the N addition effects on soil-available micronutrients because it impacts soil pH [19,36]. Although changes in micronutrients were smaller regardless of plant and soil in mown plots as compared with those in unmown plots, such effects were not significant in most cases, especially in the three plant parts. A possible reason might be that mowing management counteracted the increasing effects of N addition on plant micronutrients by weakening their uptake intensity [22,23]. Moreover, although mowing caused micronutrient loss by plant material removal, it also stimulated root growth, and the roots in mown plots with N addition could store more micronutrients, suggesting that plants alter their nutrient use strategies. However, we found that soil micronutrients were lost more under conjunct N addition and mowing treatment as compared with those under individual N addition or mown treatment. Two reasons might help explain this point. First, in N addition plots, mowing could mitigate the negative effects of N addition on plant growth, such as avoiding NH^4+^ and Mn^2+^ toxicity, and alleviate the competitive advantage of some nitrophilous plant species [43,44,45]. Thus, plants absorb more micronutrients, and the removal of plant biomass and micronutrients could reduce the accumulation of micronutrients in soil. Second, mowing could alter abiotic factors, such as by increasing light availability or decreasing soil water preservation [42,46]. In this respect, the binding force of soil micronutrients with humus and clay minerals would become weaker and more micronutrients might be lost via leaching [47,48]. Additionally, we found that both soil total and available Fe were significantly correlated with Fe in both aboveground plant, litter and roots, with no similar correlation for the other five micronutrients under observation (Figure 6 and Figure S5). This is on account of the disproportionate uptake of micronutrients by plants and an inconsistent distribution of these micronutrients in different plant parts [1,3].

In the present study, we quantized systematically the responses of six micronutrients, including two often neglected elements, Co and Mo, to long term N and mowing in the plant and soil system. We also highlighted the effects of the two extrinsic factors of micronutrient redistribution on the plant and soil responses to micronutrient toxicity and deficiencies. Nevertheless, research in the future could be conducted in regard to the following three aspects. First, the underlying mechanisms for the uptake and allocation of micronutrients by plants based on their need to help improve the regulation of micronutrients in grassland ecosystems should be examined [49,50]. Second, the relationships of soil micronutrient content with plant uptake under N addition and mowing should be elucidated [50]. Finally, whether changes in micronutrient content in plants impact forage quality and livestock health in pasture systems need to be assessed [3,29,51].

## 5. Conclusions

Nitrogen addition and mowing significantly interacted to affect the contents and pools of micronutrients in both plants and soil, but the overall effects of N addition were stronger than those of mowing especially in the three plant parts (i.e., aboveground plant parts, litter and roots). N addition increased the contents of six soil-available micronutrients but decreased the pools of soil total micronutrients. In contrast, although the overall effects of N addition on micronutrients were positive (increasing effects), such effects were generally significant under N10 and N50 treatments. Notably, litter and roots stored more micronutrients after long term N addition and mowing management. Significant positive correlations among these six micronutrients were observed in the plant–soil system, except for Fe with Mn in aboveground plants and litter. These findings can be considered in models of the nutrient biogeochemical cycling when assessing the possible effects of land management changes and N fertilization in pasture ecosystems.

## Data Availability

The data presented in this study are available on request from the corresponding author. The data are not publicly available due to privacy.

## Supplementary Materials

The following supporting information can be downloaded at: https://www.mdpi.com/article/10.3390/plants11223042/s1, Table S1: Effects of nitrogen addition on soil pH and soil organic carbon (SOC) content with unmowing and mowing treatments; Figure S1: Effects of N addition on the contents of six soil total micronutrients with unmowing and mowing treatments; Figure S2: Effects of N addition on the pools (dry biomass × contents) of six micronutrients with unmowing and mowing treatments in aboveground plant parts; Figure S3: Effects of N addition on the contents of six micronutrients with unmowing treatments in litter; Figure S4: Effects of N addition on the pools of six micronutrients with unmowing and mowing treatments in belowground root; Figure S5: Relationships of soil total Fe, Mn, Cu, Zu, Co, and Mo with their corresponding contents inaboveground plants (purple points), litter (green triangles) and roots (orange points).
