# Supplementary material for: Effects of 12-Year Nitrogen Addition and Mowing on Plant-Soil Micronutrients in a Typical Steppe
Source: Plants (Basel). 2022 Nov 10;11(22):3042. doi: 10.3390/plants11223042 (PMC9697658; doi:10.3390/plants11223042)

Supporting materials

Table S1 Effects of nitrogen addition on soil pH and soil organic carbon (SOC) content with unmowing and mowing treatments. Notes, Different lowercase letters denoted significant differences (p < 0.05) between control and N addition plots with unmowing and mowing treatments.

		Control	N2	N10	N50
pH	Unmowing	7.22(0.05)a	7.54(0.28)a	5.75(0.50)b	4.31(0.10)c
	Mowing	7.12(0.25)a	7.25(0.31)a	6.52(0.47)b	5.08(0.14)c
SOC	Unmowing	20.84(0.50)b	20.74(0.25)b	27.01(1.78)a	25.95(1.39)a
	Mowing	21.91(1.13)b	24.77(0.97)a	25.68(0.40)a	23.37(1.75)ab

Figure S1. Effects of N addition on the contents of six soil total micronutrients with unmowing and mowing treatments. Notes: Data were shown as means ± standard error. The results of two-way ANOVA were shown at the top of each figure. *, **, *** indicate significant differences at $p <$ 0.05, 0.01, 0.001, respectively (F values). Different capital or lowercase letters denote significant differences ($p < 0.05$) between control and N addition plots with unmowing and mowing treatments. Symbols +, -indicate significant positive or negative effects of mowing compared to their counterparts with the same N addition rates at $p < 0.05$.

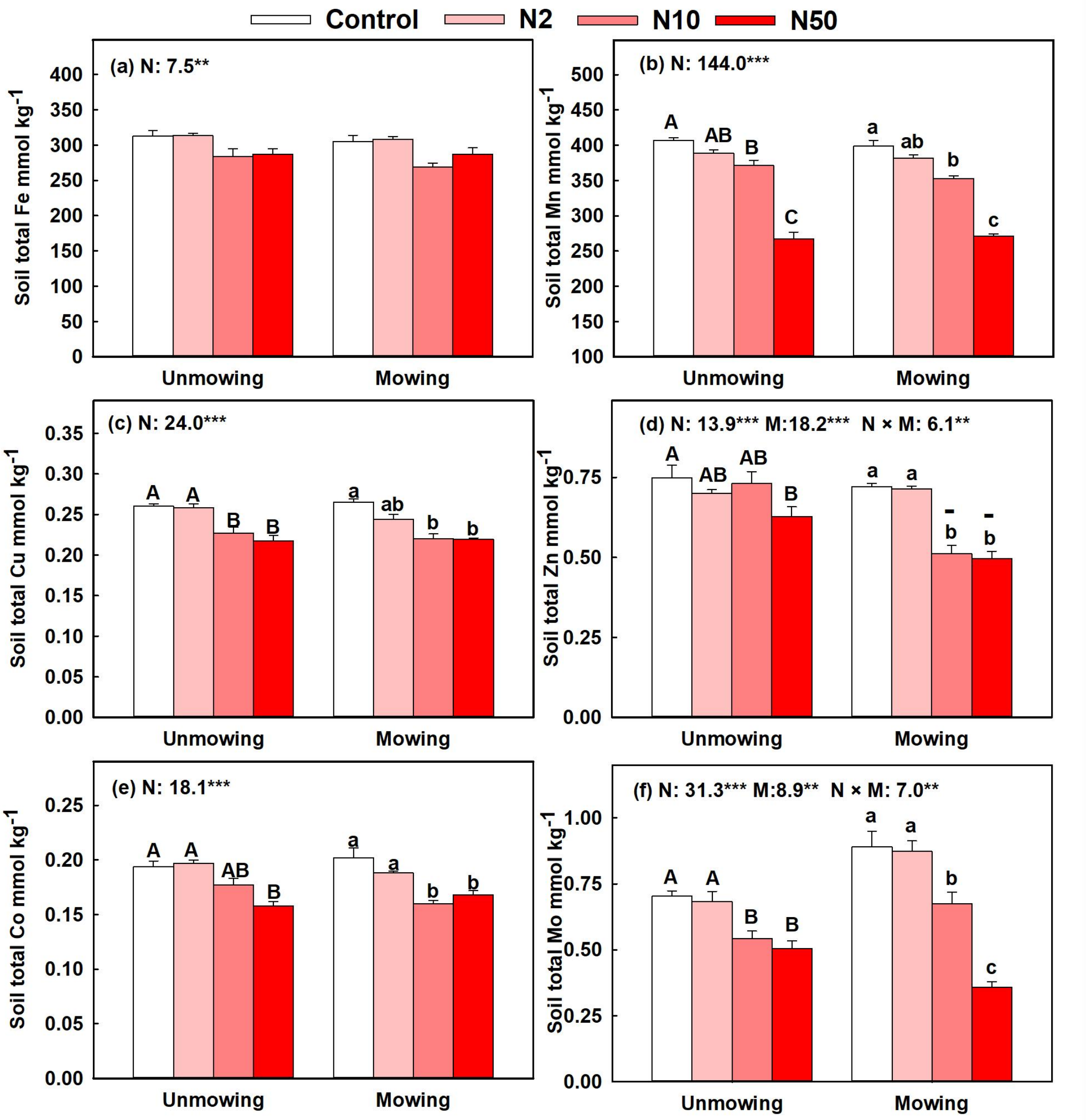

Figure S2. Effects of N addition on the pools (drybiomass × contents) of six micronutrients with unmowing and mowing treatments in aboveground plant parts. See notes in Fig. S1.

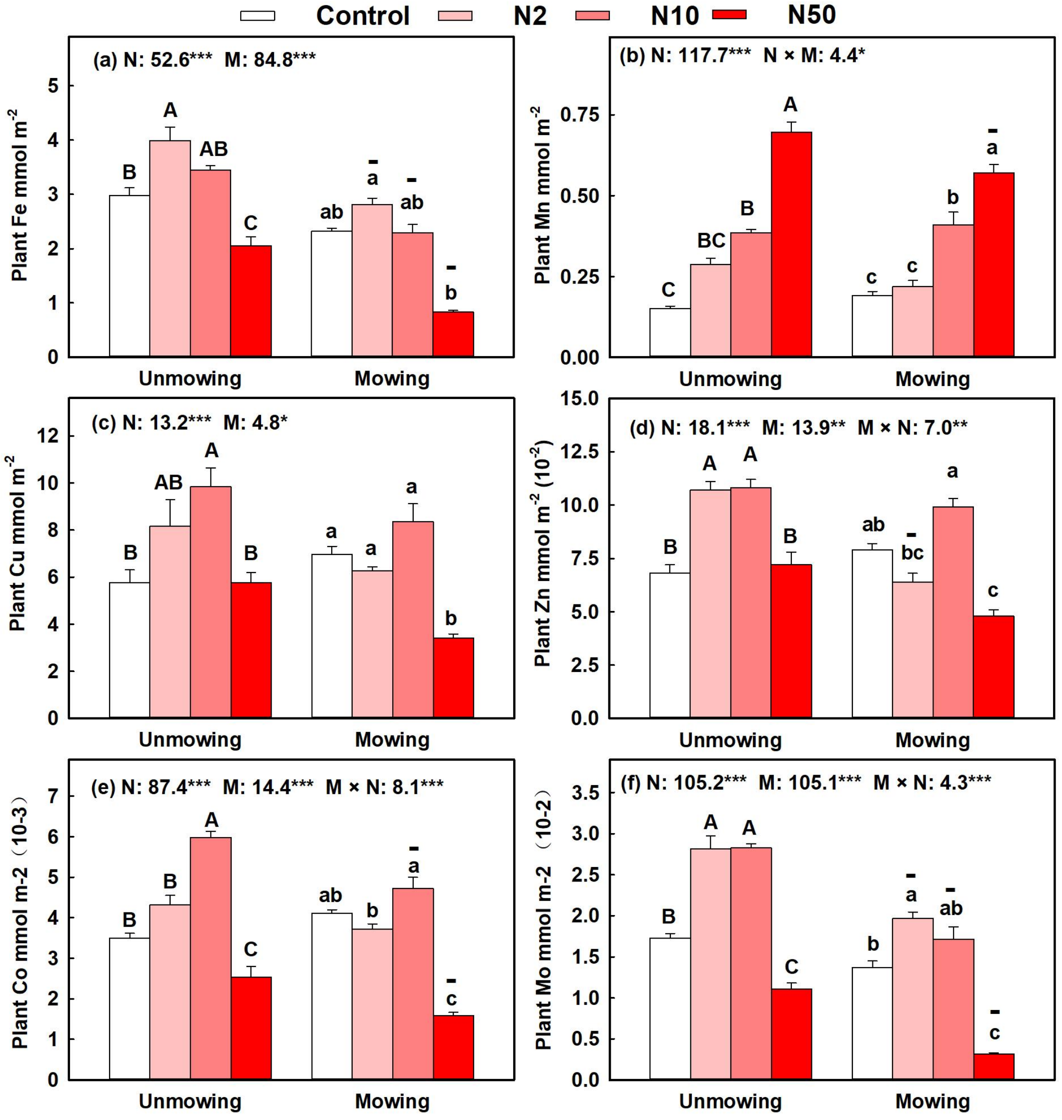

Figure S3. Effects of N addition on the contents of six micronutrients with unmowing treatments in litter. Notes: Different capital letters denote significant differences ($p < 0.05$) between control and N addition plots.

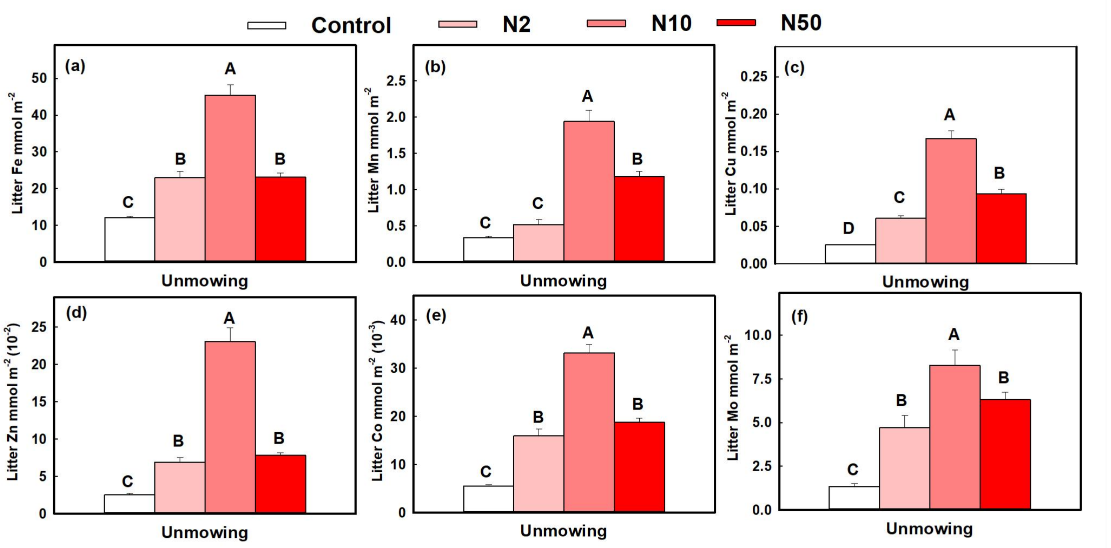

Figure S4. Effects of N addition on the pools of six micronutrients with unmowing and mowing treatments in belowground roots. See notes in Fig. S1.

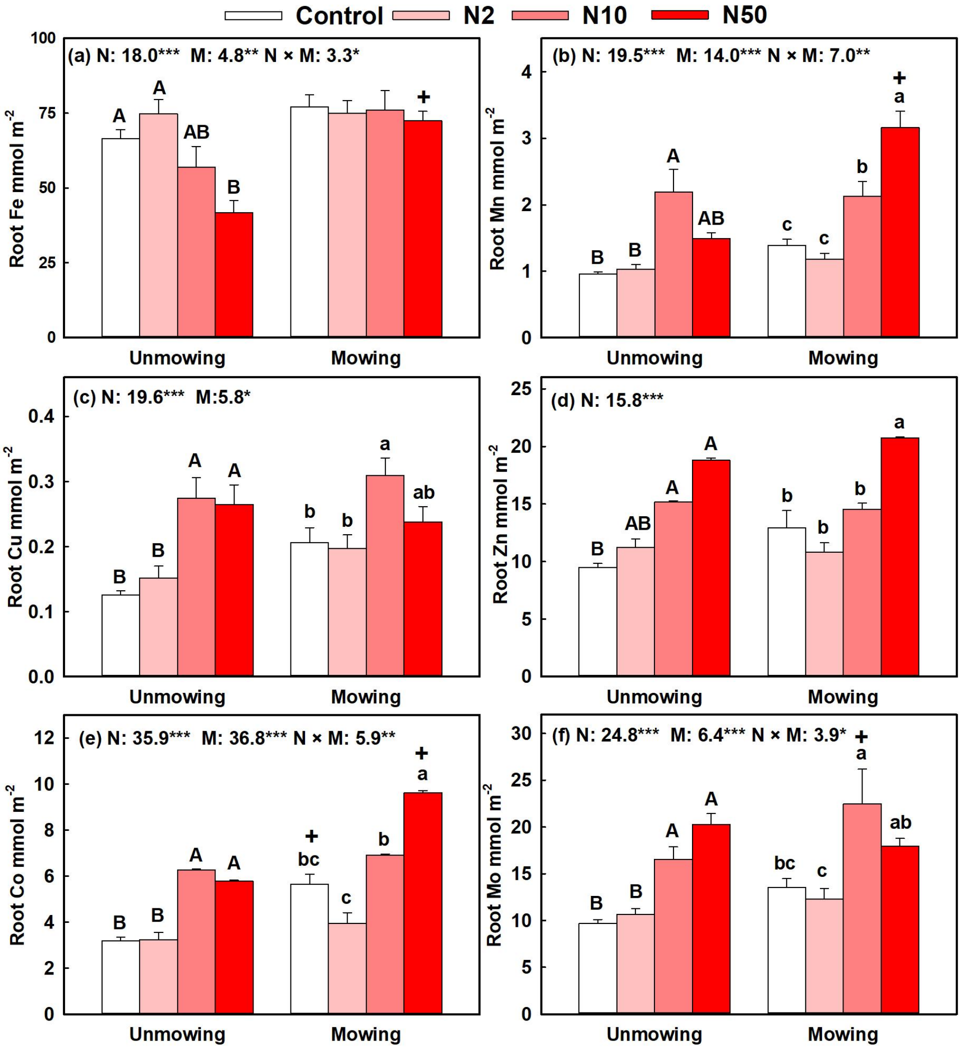

Figure S5. Relationships of soil total Fe, Mn, Cu, Zu, Co, and Mo with their corresponding contents in aboveground plants (purple points), litter (green triangles) and roots (orange points). Notes: The results (R2 and p value) of linear regression were shown at the top of each figure using corresponding color and symbols. Only significant items were shown.

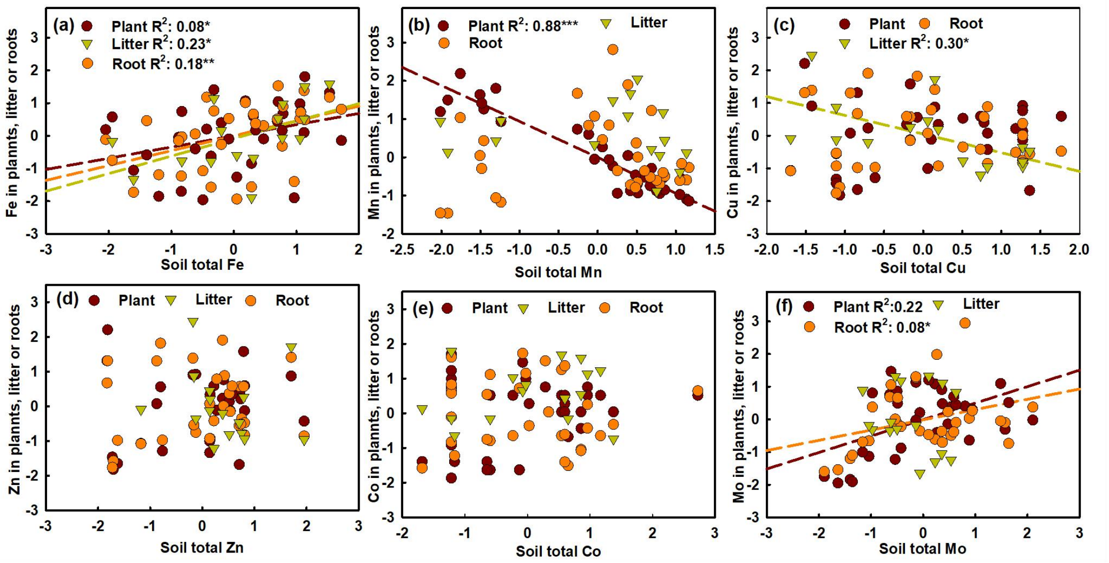

Supplement: Supplementary file 1 [file plants-11-03042-s001.zip › plants-1982589-supplementary.pdf]
